# Editorial: Exploring immune evasion and vaccine strategies in host-pathogen interactions

**DOI:** 10.3389/fimmu.2026.1828398

**Published:** 2026-04-14

**Authors:** Venkatesh Kumaresan, Rajesh Palanisamy, Pitchiah Sivaperumal, Prasanth Bhatt

**Affiliations:** 1 Department of Molecular Microbiology and Immunology, The University of Texas at San Antonio, San Antonio, TX, United States; 2 South Texas Center for Emerging Infectious Diseases, San Antonio, TX, United States; 3 Department of Immunology and Molecular Microbiology, Texas Tech University Health Sciences Center, Lubbock, TX, United States; 4 Centre for Marine and Aquatic Research (CMAR), Saveetha Institute of Medical and Technical Sciences, Saveetha University, Chennai, Tamil Nadu, India; 5 Department of Marine Sciences, Saveetha Institute of Natural and Physical Sciences, Saveetha Institute of Medical and Technical Sciences, Saveetha University, Chennai, Tamil Nadu, India; 6 Department of Biotechnology, Faculty of Science and Humanities, SRM Institute of Science and Technology, Kattankulathur, Tamil Nadu, India

**Keywords:** epitope designing and screening, host-pathogen interaction, host-targeted therapies, immunoinformatic, mucosal adjuvant, vaccines

Interaction between hosts and pathogens is a dynamic evolutionary race in which pathogens continuously evolve mechanisms to evade immune recognition while the host immune system adapts to detect and eliminate invading microbes. Understanding these processes has become increasingly critical for the development of next-generation vaccines and immunotherapies. Advancement in technologies allowed us to understand the overview of how pathogens manipulate host immune pathways can reveal novel opportunities for vaccine design. For example, studies on the intracellular pathogen *Coxiella burnetii* have demonstrated that virulent and avirulent strains differentially manipulate neutrophil autophagy, highlighting how subtle variations in pathogen virulence factors can reshape innate immune responses to favor pathogen persistence (Kumaresan et al.). Importantly, these mechanistic insights have also informed the evaluation of avirulent strains as potential live attenuated vaccine candidates, illustrating how dissecting immune evasion strategies can directly translate into rational approaches for vaccine development (Kumaresan et al.) (Palanisamy et al.). The articles compiled in this Research Topic *“Exploring Immune Evasion and Vaccine Strategies in Host-Pathogen Interactions”* provide a comprehensive perspective on emerging strategies that pathogens use to evade immunity and how innovative vaccine technologies can counteract these mechanisms. Collectively, the studies under this topic cover viral, bacterial, and host-targeted diseases involving approaches ranging from molecular immunology and structural biology to immunoinformatics and experimental immunology.

Among the common problems associated with vaccine design, the central one is the antigen design and epitope targeting in overcoming immune evasion. Pathogens frequently evade immunity by altering antigenic structures or masking immunodominant epitopes. The review examining glycosylation-mediated immune evasion in measles and mumps viruses highlights how host-derived glycosylation modifications of viral surface glycoproteins reshape antigenic landscapes and influence both innate and adaptive immune responses. Variations in glycosylation patterns within the MeV-H, MeV-F, MuV-HN, and MuV-F proteins may alter epitope accessibility and affect vaccine-induced immunity (Galvan et al.). These findings underscore the importance of accounting for post-translational modifications when designing improved vaccine strains or therapeutics.

Adding to this vaccine epitope perspective, several original research studies demonstrate how precision epitope engineering can circumvent immune escape mechanisms. Using an immunoinformatics-guided approach, Yu et al. designed a multi-epitope chimeric vaccine targeting the emerging pathogen *Mycoplasma phocimorsus*, an increasingly recognized cause of bloodstream infections and sepsis. By integrating subtractive genomics, antigenicity screening, and molecular modeling, the authors identified epitopes from a conserved immunoglobulin-blocking outer membrane protein capable of stimulating both innate and adaptive immune responses through Toll-like receptor-4 engagement. This work illustrates how computational vaccine design can accelerate vaccine discovery against emerging or neglected pathogens.

Another study from Luo et al. expands the concept of precision antigen targeting through the development of a vaccine directed against the sclerostin protein, a negative regulator of bone formation implicated in osteoporosis. By identifying a key epitope within the sclerostin loop domain and engineering a vaccine construct fused with a diphtheria toxin translocation domain, the investigators generated an immunogen capable of eliciting strong antibody responses and modulating bone remodeling pathways. This work highlights the expanding application of vaccine technologies beyond infectious diseases toward therapeutic vaccination against chronic non-infectious disorders.

Several contributions also address the challenge of inducing broadly protective immunity against highly variable viruses. Influenza viruses and coronaviruses exemplify pathogens that rapidly evolve antigenic variants, limiting the durability of conventional vaccines. Sop et al. studied the T cell responses among laboratory workers receiving seasonal influenza vaccines and identified that although effector responses to influenza antigens were relatively modest, memory T cells capable of cross-recognizing hemagglutinin epitopes from seasonal and highly pathogenic H5N1 strains were present in many individuals. These findings emphasize the importance of targeting conserved T cell epitopes to achieve cross-protective immunity against pandemic influenza strains.

Similarly, a pan-coronavirus mucosal vaccine based on conserved T and B cell epitopes from spike, nucleocapsid, and membrane proteins demonstrated robust and durable immunity in mice (Patel and Agrawal). Intranasal immunization with the mixed lipopeptide vaccine induced systemic and mucosal antibody responses, including long-lasting IgA at mucosal surfaces and persistent memory B and T cells in the lungs, spleen, and bone marrow. The incorporation of heat-killed *Caulobacter crescentus* as a mucosal adjuvant further enhanced immune memory, highlighting the importance of adjuvant design and mucosal immunity in next-generation vaccine strategies.

Another innovative approach adopted by Walimbwa et al. to overcoming viral immune escape is illustrated in the development of mimotope-based vaccines targeting broadly neutralizing antibodies against Hepatitis C virus (HCV). By using combinatorial scaffold libraries to identify non-cognate ligands that mimic the paratopes recognized by HCV-neutralizing antibodies, investigators generated immunogens capable of inducing cross-neutralizing antibody responses against diverse HCV pseudoviruses in mice. This strategy demonstrates the potential of antibody-guided vaccine design, where immunogens are engineered to mimic epitopes recognized by potent neutralizing antibodies.

At the conceptual level, the review on fourth-generation vaccines highlights the technological foundation enabling many of these advances (Araújo et al.). Emerging vaccine platforms including synthetic mRNA, self-amplifying RNA, and nanomaterial-based delivery systems have dramatically accelerated vaccine development and expanded the possibilities for antigen design. Beyond technological innovation, the review emphasizes the importance of equitable access, particularly for neglected tropical diseases such as leishmaniasis, where vaccine development has historically lagged. The integration of regional manufacturing capacity, global collaboration, and technology transfer will be critical for translating these advances into widespread public health benefits.

Taken together, the contributions in this Research Topic reveal several converging principles shaping the future of vaccine development. First, epitope-focused vaccine design, supported by immunoinformatics and structural biology, is emerging as a powerful strategy to overcome antigenic variability and immune evasion. Second, broad protective immunity, particularly through conserved T cell epitopes and cross-reactive antibodies, is increasingly recognized as essential for combating rapidly evolving pathogens. Third, innovative delivery platforms and adjuvants, including mucosal vaccines and nanotechnology-based systems, are expanding the scope and durability of immune responses. Finally, the translation of these innovations into global health solutions will require sustained efforts to ensure equitable access to next-generation vaccines.

**Figure 1:**
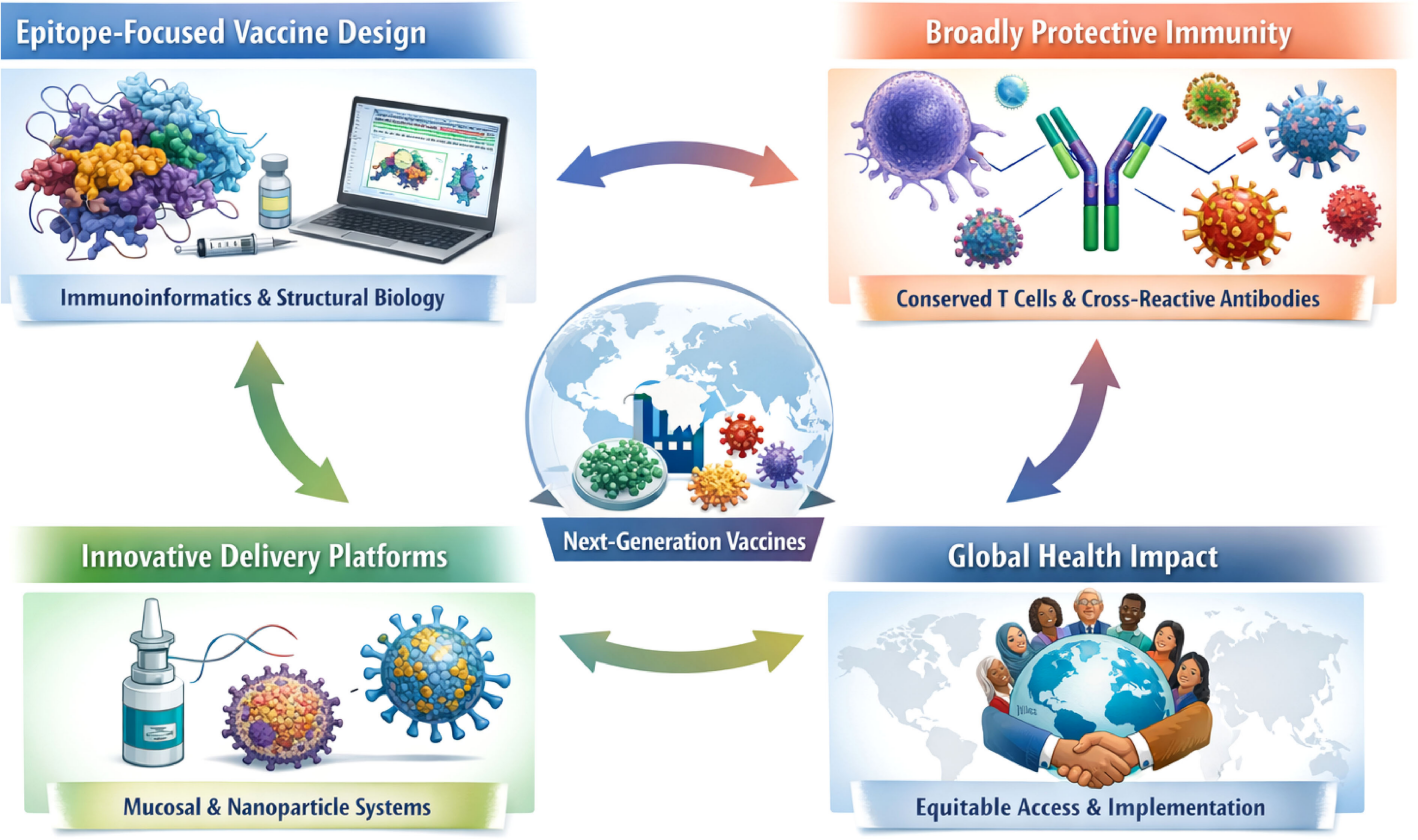
Graphical overview illustrating four interconnected themes in this Research Topic: epitope-focused vaccine design enabled by immunoinformatics; induction of broadly protective immunity through conserved T cell and antibody responses; development of innovative mucosal and nanoparticle-based delivery platforms; and advancement of global health through equitable access and effective implementation.

In summary, this Research Topic highlights the remarkable progress being made at the intersection of pathogen biology, immunology, and vaccine engineering (**Figure 1**). By integrating insights into immune evasion with advances in precision antigen design and vaccine platforms, these studies collectively move the field closer to the development of durable, broadly protective vaccines capable of addressing both emerging infectious threats and chronic diseases.

